# Evaluating the impact of nitisinone at mosquito-lethal doses on *Lutzomyia longipalpis*

**DOI:** 10.1371/journal.pntd.0013871

**Published:** 2026-01-12

**Authors:** Laure Augendre, Lucas Alexandre Farias de Souza, Magda Clara Vieira da Costa Ribeiro, Jérôme Depaquit, Jean-Philippe Martinet, Jorian Prudhomme

**Affiliations:** 1 UR ESCAPE Epidemiosurveillance and circulation of parasites in the environment, University of Reims Champagne-Ardenne, Faculty of Pharmacy, Reims, France; 2 Insects Vectors and Parasites Laboratory, Department of Basic Pathology and Postgraduate program in Microbiology, Parasitology and Pathology, Federal University of Paraná, Curitiba, Brazil; 3 Laboratoire de Parasitologie-Mycologie, Centre Hospitalo-Universitaire, Pôle de Biologie Territoriale, Reims, France; London School of Hygiene and Tropical Medicine, UNITED KINGDOM OF GREAT BRITAIN AND NORTHERN IRELAND

## Abstract

Nitisinone, a systemic inhibitor of tyrosine catabolism, has recently emerged as a promising endectocide with demonstrated lethality against mosquitoes and tsetse flies. To assess its efficacy against *Lutzomyia longipalpis*, an important vector of *Leishmania infantum* —the causative agent of zoonotic visceral leishmaniasis prevalent in the Mediterranean basin, Latin America, and parts of the Middle East—, we exposed sand flies to a concentration previously shown to be lethal to *Anopheles* mosquitoes (250 ng/mL). Survival was monitored over 14 days and showed no significant difference between treated and control groups, indicating a lack of insecticidal effect at this dose. These findings suggest that *Lu. longipalpis* exhibits tolerance to nitisinone, likely due to species-specific metabolic differences. Although nitisinone remains a promising tool for systemic vector control, its effectiveness against sand flies may require higher doses or alternative compounds.

## Author summary

We investigated whether nitisinone could help control sand flies, which are insects responsible for transmitting *Leishmania infantum*, the parasite that causes visceral leishmaniasis. Previous studies showed that nitisinone can kill mosquitoes and tsetse flies when present in treated animal blood. To test if the same approach could work for sand flies, we exposed them to a dose of nitisinone lethal for mosquitoes. Unlike mosquitoes, sand flies, after two weeks survival monitoring, were not affected by this treatment and survived as well as untreated flies. This result indicates that sand flies may be more tolerant to nitisinone, probably because of differences in their metabolism compared to other insect species. While nitisinone remains an interesting option for controlling some disease vectors, it may not be effective against sand flies at the tested dose. This highlights the need to evaluate each vector species individually and to explore other drugs or higher doses when developing systemic tools to reduce the spread of vector-borne diseases.

## Introduction

As vectors of parasitic, bacterial, and viral pathogens, phlebotomine sand flies represent a major threat to both human and animal health [1]. Vector control strategies aim to reduce or even interrupt the transmission of associated diseases [2]. Several control measures are currently available, mainly involving chemical insecticides [3]. However, their effectiveness is steadily declining due to the increasing development of insecticide resistance. Reports of sand fly resistance to organophosphates, carbamates, pyrethroids, DDT and other chemical classes have emerged in numerous endemic regions, jeopardizing the sustainability of current strategies [1,3]. In this context, vector control remains a cornerstone in the prevention and management of vector-borne pathogens [4].

More than twenty-one *Leishmania* species are recognized as pathogenic to humans [5] and their transmission by sand flies, which exhibit complex ecological behaviors, makes their control particularly difficult. They can colonize a wide array of environments — including outdoor, peridomestic, and sylvatic habitats — and often seek refuge in microhabitats inaccessible to standard intervention tools [6,7]. This high degree of ecological plasticity, coupled with challenges in implementing integrated vector management programs, significantly limits the success of existing control measures.

Given the limitations of current methods, developing new complementary strategies is crucial to reduce sand fly survival and limit disease transmission. Among the promising alternatives, endectocides — systemic agents administered to human or animal hosts — are attracting growing interest [8,9]. These compounds can exert lethal or sublethal effects on blood-feeding arthropods. Ivermectin, for example, has shown efficacy against vectors such as mosquitoes and tsetse flies, and preliminary findings indicate a similar impact on sand flies, by decreasing their survival and disrupting parasite development [10].

More recently, nitisinone — an inhibitor of 4-hydroxyphenylpyruvate dioxygenase (HPPD), approved for the treatment of hereditary tyrosinemia type I (HT-1) and alkaptonuria — has emerged as a potential systemic insecticide. By disrupting tyrosine metabolism, a process critical after a blood meal, it causes toxic accumulation of tyrosine in blood-feeding vectors such as mosquitoes, ticks, and tsetse flies [11–14]. Unlike conventional neurotoxic insecticides, nitisinone operates via a novel mode of action, making it a promising candidate for next-generation vector control.

However, its effects on the genus *Lutzomyia*, and more widely on sand flies, remain unknown. In this study, we evaluate the pharmacodynamic profile of nitisinone for sand fly control. We present proof-of-concept data to investigate whether a dose lethal to *Anopheles* mosquitoes can similarly induce significant mortality in sand flies, potentially offering an innovative vector control strategy.

## Materials and methods

### Sand flies rearing

To conduct experimental studies, we used a *Lu. longipalpis* colony originated from Jacobina (Bahia) maintained at Federal University of Paraná, Curitiba, Brazil, following previously described protocol [15]. Sand fly colonies were reared in a climate chamber under standardized conditions (26 ± 1°C, 80% relative humidity, with no light-dark cycle). Adults were provided with cotton pads soaked in a 50% organic sugar solution as a food source.

### Nitisinone preparation and administration to sand flies

Nitisinone (2-[2-nitro-4-(trifluoromethyl)benzoyl]cyclohexane-1,3-dione; Sigma Aldrich, St Louis, USA) was solubilised in 1M NaOH, and the pH was adjusted to 7.5 with 6M HCl as previously described [16]. Aliquots were prepared at a final concentration of 250 ng/mL and stored at −20 °C.

The day before the bloodmeal, *Lu. longipalpis* (4–10 days old) were randomly sorted into four groups of 150 females and 25 males in nylon mesh cages, and sugar was removed. On the day of the blood meal, each cage was provided a glass feeder containing rabbit blood (with EDTA 1%) supplemented with either 250 ng/mL nitisinone (treatment group) or phosphate-buffered saline (control group), as previously described [16,17]. The following day, only blood-fed females were selected, and unfed one were removed and discarded. Mortality was recorded daily for 14 consecutive days.

### Statistical analysis

Statistical analysis and graphics were performed using R and Rstudio software (version 2023.12.0; R Foundation for Statistical Computing, Vienna, Austria). The log-rank (Kaplan–Meier) test was used to evaluate significant differences in survival between the experimental and control groups. The log-rank (Mantel-Cox) test was used to assess survival curves. The analysis was performed using “survival” and “survminer” packages in R [18–20].

## Results

**Fig 1:**
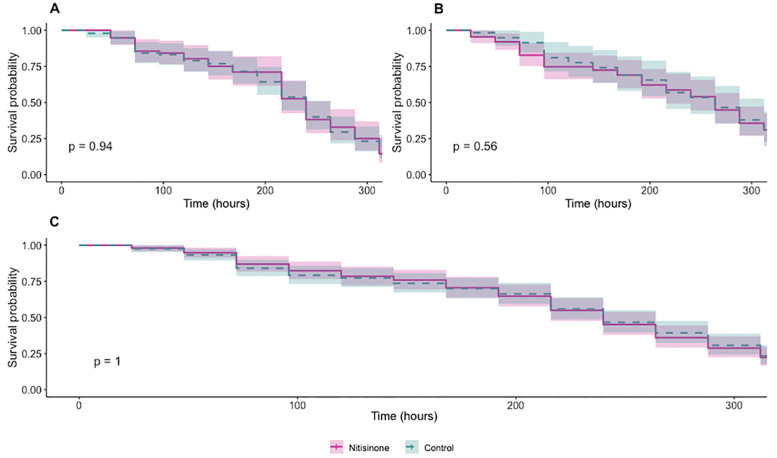
Sand flies survival after a blood meal containing nitisinone (250ng/mL; a dose lethal to mosquitoes). **(A and B)** Kaplan–Meier survival curves show two replicate experiments measuring sand flies survival over 14 days after feeding on a blood meal containing 250 ng/mL of nitisinone (purple) compared to untreated controls (blue dotted line). **(C)** Pooled data from both replicates. Statistical significance was assessed using the log-rank (Mantel–Cox) test to compare survival between treated and control groups.

To assess whether a mosquito-lethal dose of nitisinone (250 ng/mL) impacts the survival of *Lu. longipalpis*, Kaplan–Meier survival analyses were conducted over a 14-day period in two independent experimental replicates. No significant difference in survival probability was observed between treated and control groups in either replicate (log-rank test, p > 0.05; Fig 1A and 1B). In both cases, the mortality pattern was gradual and comparable across groups, with no treatment-associated increase in death. Pooled analysis (Fig 1C) confirmed these observations.

## Discussion

Our results indicate that the 250 ng/mL nitisinone concentration fails to compromise *Lu. longipalpis* survival, whereas the same dose significantly reduces survival in *Anopheles* mosquitoes [16]. This outcome could reflect a species-specific tolerance of *Lu. longipalpis* to nitisinone. Although sand flies and mosquitoes are not phylogenetically closely related, both are dipterans and share some comparable traits, such as small body size and limited blood meal volume. These similarities made the mosquito-lethal dose a reasonable starting point for initial testing in the absence of sand fly-specific data. However, the absence of a toxic effect suggests that *Lu. longipalpis*, like tsetse flies, may require higher concentrations to elicit systemic toxicity. Indeed, *Glossina* spp. also exhibit susceptibility to nitisinone, but only at substantially higher doses [11], despite their ingestion of larger blood volumes.

It is important to emphasize, however, that no positive control was included in the present study. Indeed, numerous methodological and biological differences limit the direct comparability between studied species (Mosquitoes and sand flies). Variations in experimental conditions — such as the use of different feeding devices (Hemotek *vs*. glass feeders), membranes (parafilm *vs*. chick skin), and host preferences (the strong anthropophily of *An. gambiae vs.* the opportunistic feeding of *Lu. longipalpis*) — are likely to introduce substantial variability. Moreover, differences in feeding behavior (solenophagy in mosquitoes *vs.* telmophagy in sand flies), as well as the physico-biological properties of the host skin (chick skin *vs*. artificial membrane), could affect nitisinone uptake and toxicity, thereby compromising the reliability of a cross-species positive control. Finally, our primary objective was to perform an initial exploratory assessment of nitisinone activity in *Lu. longipalpis*. Therefore, the most appropriate positive control would be *Lu. longipalpis* itself exposed to higher nitisinone concentrations.

As a 4-hydroxyphenylpyruvate dioxygenase (HPPD) inhibitor, nitisinone disrupts tyrosine degradation, leading to toxic accumulation of tyrosine post-blood meal. It is plausible that *Lu. longipalpis* sand flies possesses compensatory metabolic mechanisms or tissue-specific sequestration strategies that limit the toxicity of accumulated tyrosine—mechanisms that may be absent or less effective in *Anopheles* mosquitoes. The absence of nitisinone effect could reflect interspecific differences in tyrosine metabolism or detoxification pathways.

Supporting the importance of species-specific differences, no impact on the survival of *Glossina pallidipes* at 250 ng/mL of nitisinone were reported [11]. However, more than 50% of treated *G. pallidipes* individuals died within 20 hours when exposed to concentrations equal to or exceeding 500 ng/mL, suggesting a sharp threshold for toxicity in this species. These results further underscore the importance of dose optimization and species-specific physiological traits when evaluating systemic endectocides for vector control. These factors alone are unlikely to account for the results. Thus, detoxification capacity and physiological resilience may be more critical determinants of species-specific sensitivity.

Finally, *in vivo* evaluation of this molecule in a host organism will be critical, as pharmacokinetic factors may influence efficiency and the development of resistance. Differences in absorption, distribution, metabolism, or excretion of nitisinone could reduce its systemic bioavailability in sand flies. Even if an effective dose is identified under laboratory conditions, its relevance must be re-evaluated considering pharmacokinetics, as laboratory settings cannot fully replicate natural feeding behaviors (e.g., no natural telmophagy mechanisms in artificial feeding settings). Indeed, feeding behavior may influence drug uptake, as *Lu. longipalpis* are telmophagous (lacerating host tissues to create a blood pool) whereas mosquitoes are solenophagous (puncturing blood vessels). The mechanical nature of telmophagy may result in a different composition of ingested fluids, potentially diluting the concentration of systemically circulating drugs like nitisinone in the bloodmeal. Moreover, the ingestion of interstitial fluid, damaged cell contents, and clotting factors could affect the stability or absorption of the drug within the insect midgut. In contrast, solenophagous vectors may ingest more concentrated plasma directly from the bloodstream, possibly leading to more consistent and higher drug uptake.

Nitisinone remains a promising systemic endectocide for mosquito control. However, its efficacy at mosquito-lethal concentrations appears limited against *Lu. longipalpis*. This highlights the necessity of species-specific assessment in the development of pharmacological vector control strategies. Future research should aim to characterize dose-response relationships, quantify internal drug concentrations, and investigate the pharmacodynamics of HPPD inhibition in sand flies. Such studies will be essential to determine whether higher, safely tolerated doses of nitisinone—or alternative compounds—could be effectively leveraged for integrated control of sand fly vectors. Finally, future studies should evaluate whether nitisinone impacts *Leishmania* development within *Lu. longipalpis*, as ivermectin has been reported to interfere not only with vector survival but also with parasite development [10]. Understanding whether nitisinone can similarly disrupt parasite transmission could broaden its utility, even in the absence of direct insecticidal effects at lower concentrations.

## Conclusions

Our preliminary findings demonstrate that *Lu. longipalpis* sand flies exhibit a marked tolerance to nitisinone at concentrations previously shown to be lethal for *Anopheles* mosquitoes. Despite the promising potential of nitisinone as a systemic endectocide for controlling insect vector, its lack of efficacy against sand flies at 250 ng/mL highlights the critical need for species-specific assessments when developing drug-based vector control strategies. Rather than reflecting genuine tolerance, the limited response observed here may result from methodological constraints, physiological particularities of *Lu. longipalpis*, or the need for an alternative experimental design to capture dose-dependent effects. Future dose–response studies, including appropriate intra-species controls, will be crucial to determine whether sand flies are inherently less sensitive to nitisinone or whether exposure levels were insufficient to elicit toxicity.

These findings highlight the complexity of translating systemic insecticides across vector taxa and suggests that the metabolic or physiological features of *Lu. longipalpis* may render them less susceptible to disruption of tyrosine catabolism. Further research is necessary to determine whether higher doses of nitisinone could improve efficacy against sand flies, or whether alternative HPPD inhibitors may offer greater promise.

In the broader context of integrated vector management, our results emphasize that reliance on a single class of systemic agents is unlikely for universal solutions. Instead, customized approaches tailored to the ecological and biological characteristics of each target vector will be essential for achieving sustainable and broad-spectrum vector control.
